# Good safety practice in a randomized controlled trial (CadColdEx) involving increased cardiac workload in patients with coronary artery disease

**DOI:** 10.1186/s12872-019-1051-1

**Published:** 2019-03-25

**Authors:** Tiina M. Ikäheimo, Miia Länsitie, Rasmus Valtonen, Heidi E. Hintsala, Niilo Ryti, Juha Perkiömäki, Matti Mäntysaari, Arto J. Hautala, Jouni J. K. Jaakkola

**Affiliations:** 1 Center for Environmental and Respiratory Health Research, University of Oulu, P.O. Box 5000, FI-90014 Oulu, Finland; 2 Medical Research Center Oulu, Oulu University Hospital and University of Oulu, FI-90014 Oulu, Finland; 3 Research Unit of Internal Medicine, University of Oulu, FI-90014 Oulu, Finland; 4 The Finnish Defence Forces, Aeromedical Centre, FI-00290 Helsinki, Finland; 5 Cardiovascular Research Group, Division of Cardiology, Oulu University Hospital, University of Oulu, Oulu, Finland

**Keywords:** Controlled experiment, Patient safety, Coronary artery disease, Exercise, Cold temperature

## Abstract

**Background:**

Methodological information acknowledging safety of cardiac patients in controlled medical experiments are lacking. The descriptive report presents one good practice for considering safety in a randomized controlled study involving augmented cardiovascular strain among persons with coronary artery disease (CAD).

**Methods:**

The patients were pre-selected by a cardiologist according to strictly defined selection criteria. Further confirmation of eligibility included screening of health. In addition, assessments of physical capacity by a graded bicycle ergometer test were implemented and safety monitored by an exercise physiologist and medical doctor. In this context, an emergency simulation was also carried out. A total of 18 CAD patients each underwent four different experimental interventions where either temperature (+ 22 °C and − 15 °C) and the level of exercise (rest and brisk walking) were employed for 30 min in random order (72 experiments). Baseline (20 min) and follow-up (60 min) measurements were conducted resting at + 22 °C. ECG, and brachial blood pressure were measured and perceived exertion and symptoms of chest pain inquired throughout the experiments. An emergency nurse was responsible for the health monitoring and at least two persons followed the patient throughout the experiment. A medical doctor was available on call for consultation. The termination criteria followed the generally accepted international guidelines for exercise testing and were planned prior to the experiments.

**Results:**

The exercise test simulation revealed risks requiring changes in the study design and emergency response. The cardiovascular responses of the controlled trials were related to irregular HR, ST-depression or post-exercise hypotension. These were expected and the majority could be dealt on site by the research personnel and on call consultation. Only one patient was encouraged to seek for external health care consultation.

**Conclusions:**

Appropriate prospective design is a key to safe implementation of controlled studies involving cardiac patients and stimulation of cardiovascular function. This includes careful selection of participants, sufficient and knowledgeable staff, as well as identifying possible emergency situations and the required responses.

**Trial registration:**

ClinicalTrials ID: NCT02855905.

## Background

The ethical guidelines for medical research involving human subjects require careful beforehand assessment of predictable risks and burdens to the individuals involved in the study. Furthermore, measures to minimize risks must be implemented. These risks shall then be continuously monitored, assessed and documented by the researcher. In the case of vulnerable populations, protection from possible adverse health effects should specifically be considered [1].

Registries of clinical trials, such as the one maintained by the U.S. National Library of Medicine [2] provide summaries of study protocols, including information on the studied disease or condition, type of intervention, requirements for participation, outcomes of the study, and even a summary of adverse events experiences by the study participants. However, these do not have a special emphasis on describing procedures of patient safety. Experiences of appropriate planning, and steps for implementing safety at the various phases of the research protocol would be useful for researchers. To our knowledge, there are no descriptions for good safety practices of controlled studies involving cardiac patients. Any measures involving increased cardiac workload could involve a heightened risk of adverse health events among this vulnerable population. In this respect, only safety of cardiopulmonary exercise testing has been reported separately for persons with cardiovascular diseases [3].

A low environmental temperature itself increases cardiovascular strain that is manifested, for example, as a substantially higher aortic blood pressure [4] and higher morbidity and mortality particularly due to cardiovascular reasons [5]. In addition, exercise in the cold may further augment cardiac workload and result in adverse cardiac health events [6]. Patients with CAD have a reduced myocardial oxygen supply that may be insufficient to meet the higher cardiac workload and oxygen demand of both cold and exercise [7, 8]. The subsequent myocardial ischemia may lead to angina pectoris, and in the worst case, progress to myocardial infarction, fatal arrhythmias, and sudden cardiac deaths [9]. Exercise-based rehabilitation has been shown to reduce hospitalizations and cardiovascular mortality among those with coronary heart disease [10]. Therefore, the rationale was to investigate whether sustained moderate-intensity exercise recommended for the treatment of CAD patients [11] is safe when carried out in a cold environment. To our knowledge, there are no previous information of safety practices related to planning, preparing and monitoring of a controlled study as ours. The objective of the present study was to describe, in detail, the safety measures and outcomes during the different phases of a controlled study involving cardiac loading due to both exercise and cold temperature in patients with coronary artery disease (CAD).

## Methods

### Study design

The study is a randomized controlled study [12] which is registered and the design described in the ClinicalTrials (www.clinicaltrials.gov, ID: NCT02855905). The overall design, activities and responsibilities related to the study are described in Table 1. As a part of the broader research entity, we present here the implementation and outcome of patient safety as a descriptive study largely utilizing the method of observation in a controlled setting as a data collection tool.

**Table 1:** Study design, activities, phases and responsible persons

Activity	Phase of the project	Responsible person
Determining eligibility for participation, agreeing of the study protocol and required safety measures	Planning	Research team
Employing agreed selection criteria	Selection of patients from medical records	Cardiologist
Confirmation of eligibility	Contact with patients: pre-screening for eligibility, inquiring of willingness to receive further information	Cardiologist
Confirmation of eligibility	Contact with patients (*n* = 22): informing of the study, screening of health through a questionnaire, invitation to participate to tests assessing physical capacity	Researcher
Confirmation of eligibility, use of exercise information to the experiments, employing termination criteria, constant monitoring	Assessment of physical capacity (*n* = 20), graded bicycle ergometer test, measurement of ECG, acquiring informed consent to participate to the experimental study	Researchers, expert in exercise physiology, medical doctor (on site)
Planning of experiment, employing agreed termination criteria, constant health monitoring	Randomised controlled study (*n* = 18 × 4): exercise or rest at − 15 °C or + 22 °C (baseline, exercise, follow-up), assessment of cardiovascular function (HR, ECG, BP), perceived exertion, thermal sensations, assessment of possible other symptoms	Researchers, emergency nurse, medical doctor (monitoring on call)
Post-experiment follow-up	Follow-up after the experiment (*n* = 18), 24 h monitoring of BP and ECG	Researcher, participant

### Ethics approval and consent to participate

The Osthrobothnia Hospital District in Oulu, northern Finland, provided ethics approval for the study. Prior to the controlled experiments the patients received information of the study, after which they provided written informed consent to participate.

### Study population

Prior to the recruitment of participants the research team carefully planned and determined the criteria for eligibility. The following inclusion criteria were applied: male gender, CAD with a Canadian Cardiovascular Society grading of angina pectoris (CCS) class I or II, non-ST-elevation myocardial infarction (NSTEMI) more than three months before the experiments. Patients with CAD and hypertension, but no other cardiovascular, respiratory or metabolic diseases were considered eligible. The exclusion criteria were CCS class III-IV, myocardial infarction less than 3 months ago, having had a bypass surgery or cardiac pacemaker, claudication, left ventricular ejection fraction less than 40%, presenting abnormal electrocardiogram (ECG) findings, or being a current smoker. The cardiologist identified subjects with CAD and myocardial infarction from the patient registry of the Oulu University Hospital (2014–2016) and inquired of their willingness to participate to the study. Based on additional telephone screening of 28 candidates, the researchers invited 22 participants for the assessment of physical capacity. Two participants were excluded from the controlled experiments due to miscellaneous reasons (smoking, leg infection). From the 20 patients participating in the controlled experiments, two persons decided to discontinue. Hence, the final data consists of 18 persons that participated to all four interventions (*n* = 72). The patient characteristics of the study population were: age 60.1 ± 7.2 yrs., height 173.9 ± 5.2, weight 89.7 ± 18.6, BMI 29.5 ± 5.5, fat 26.5 ± 7.6%, peak VO2 ml/min/kg 29.6 ± 5.6, 89% with hypertension. More details of the participants is presented by Valtonen et al. [12].

### Intervention

#### Safety measures taking place prior to the controlled experiments

A bicycle ergometer test was conducted to assess the maximal exercise capacity of the patients and to detect possible ECG-abnormalities indicating cardiac oxygen deficit during exercise (Table 1). The termination criteria followed the AHA Scientific statement for exercise testing [13].

According to the statement of the American Heart Association the role of the physician is to ensure quality and safety of the exercise tests [14]. This includes for example regular rehearsing and documenting capacity for an emergency response. Although there were no contra-indication for a bicycle-ergometer exercise test for the participants, we implemented extended safety measures to minimize the risks and to train the staff for the experiments. A simplified form of British Standard 8800 risk assessment tool was applied [15] in the laboratory environment to identify any factors that might compromise life support, team work, and crisis resource management (CRM) [16]. The medical doctor (MD) executed an emergency simulation (a sudden severe chest pain during the ergometer test, collapse, and VF simulating a plaque rupture during the test) for the staff members (researchers) that would be monitoring the experiments, with an acting healthy volunteer wearing the exact equipment used in the study was acting as a patient receiving detailed instructions. The staff members were not aware of the type of emergency beforehand. Staff responses were observed, and problems in communication and CRM were recorded, discussed, and transformed into later safety protocols. Before the actual experiments, Life Support Scenarios and staff member-specific tasks were orally repeated as team effort.

The actual exercise tests were carried out by an exercise physiologist experienced with cardiac patients, and were monitored onsite by a MD. Based on normal blood pressure (BP) response (SBP < 260 mmHg) and ECG-finding during the test the MD further confirmed the eligibility of the subjects. The obtained fitness information was used to calculate an individually based walking speed of moderate intensity that was utilized in the experiments. The following formula was used: HRrest+ 0,45xHRR, where HR represents heart rate at rest and HRR heart rate reserve (maximal HR-resting HR) [17].

Prior to the controlled experiments the patients filled in a health questionnaire which inquired of their general state of health, health behavior, medications, as well as possible symptoms emerging during environmental cold exposure. An emergency nurse further interviewed the participants focusing on possible symptoms (e.g. chest pain, arrhythmias) occurring either during exercise or cold exposure. If the participants reported symptoms related to CAD, a MD was consulted and patients were referred, if necessary, to a medical check-up. The participants were instructed to continue their normal medication, inform of any merging infections, as well as abstain from exceptionally heavy exercise, use of alcohol for 24 h prior to the experiments.

### Intervention

#### Safety monitoring during the experimental exposure to cold temperature and exercise

Each of the patients participated in four experimental conditions where either temperature (+ 22 °C or − 15 °C) and the level of physical activity (rest or exercise) were employed in random order. The controlled experiments consisted of baseline (20 min, + 22 °C, light clothing), experimental (30 min walk or rest in + 22 °C with light clothing or at − 15 °C with winter clothing) and follow-up (60 min in baseline conditions). The assessments of cardiovascular function were carried out in two climatic chambers (Oulu, Finland) with a possibility to adjust temperature and level of exercise. Of the 20 selected subjects 18 completed all experimental conditions (total of 72 measurements) and each of these lasted for ca. three hours. During the experiments the participants were equipped with online ECG monitoring (CardioSoft, GE HealthCare, Germany) followed throughout the experiment. Automated auscultatory brachial BP measurements (Schiller BP-200 Plus, Schiller, Switzerland) were performed at 5-min intervals during the experiment. Occurrence of chest pain and perceived exertion was inquired according to a scale from 6 to 20 while exercising [18]. In addition, the general condition and skin color of the subjects was monitored and the participants were instructed to inform the researchers immediately of any deviations in their physical condition. The treadmill could be stopped immediately in any emergency situations. An emergency nurse was responsible for the health monitoring and at least two persons followed the patient throughout the experiment. An MD was available on call for consultation.

The termination criteria were agreed together with the MD and followed the generally accepted international guidelines for exercise testing and prescription [19]. The following criteria were employed: moderate, strong or increasing chest pain, nausea, dizziness, pale or bluish skin color, cold peripheral areas or cold sweat, drop of systolic BP ≥40 mmHg during the employed moderate exercise, ventricular tachycardia (4 beats or longer or several 3 beats episodes) or related to symptoms, supraventricular tachycardia, bradycardia (HR unresponsive to exercise, drop of HR despite of increase in exercise but not related to the start of exercise), ST elevation of ≥1 mm and -ST depression ≥2 mm from baseline in chest electrodes, strong dyspnea, aching feet, claudication, increase in BP > 260/ 115 mmHg, strong headache, participant wants to terminate the experiment. The participants were guided to a lying position on a bed located in the experimental facility in the case when post-exercise hypotension caused symptoms and when systolic BP was less than < 100 (< 90 mmHg), or there were any other abnormalities in their condition.

### Follow-up in the laboratory and at home

Data on cardiovascular function (ECG, BP) of the participants was collected for 60 min in the laboratory following the experimental intervention, during which their general state of health was inquired regularly. After the measurements the participants were allowed to stay based on their preference for a sufficient period of time at the research facility before leaving home. Ambulatory BP and ECG was monitored by the participants at home for 24 h following the exercise interventions. This was carried out mainly because of collecting research data, but also enabled to confirm that the participants recovered from the experiments.

## Results

### Testing of physical capacity

We decided that regardless of the fact that there was an advanced life support (ALS) provider present, the level of care in cardiac arrest would be Basic Life Support (BLS). The following factors influenced this decision: a) early defibrillation was recognized a major objective; b) we wanted to minimize the risk of errors and delays in care due to over-tasking of individuals; c) ALS providers of emergency medical services (EMS) would be available in the scene within 5 min from the emergency call. We confirmed the latter in the planning phase of the study by being in contact with the local EMS center. Therefore, our safety protocol strategically focused on a) ensuring immediate and reliable identification of cardiac arrest; b) early defibrillation; c) high-quality CPR, d) early call for help, and securing unrestricted access of the EMS providers to the scene. We also coordinated with the local EMS center that in a case of a deteriorating patient not yet in cardiac arrest, early call would be appropriate and the center would send a Medical Emergency Team (MET). Aspirin and sublingual nitrates were available according to the national protocol.

The safety simulation revealed several factors, which, if left unattended, might compromise undisturbed emergency response (Table 2). It also confirmed several factors predicted by the risk assessment tool [15]. For example, the equipment cords got tangled if the patient fell to the wrong side of the ergometer. This initiated a cascade where the experimenter assessing the patient could not get a visual of the ECG monitor. These observations lead to changes in the overall setup of the exercise equipment, staff placement, and emergency task allocation. Finally, each staff member was provided with personal written emergency instructions to ensure undisturbed and coordinated effort in critical circumstances.

**Table 2:** Problems of effective workflow and pertinent changes in the safety protocol during the simulation

Problems observed during the simulation	Resulting changes in the safety protocol
If patient collapsed to the left side of ergometer, the placement of furniture prohibited unobstructed access to the patient.	Preferable treatment area designated. Relocation of furniture and equipment. Plan B discussed.
If patient collapsed to the right side of the ergometer, monitor cords tangled around it. These had to be temporarily detached, which lead to loss of monitoring during the emergency.	Monitor cords were appropriately bundled, elevated with an adapter, and fed to the monitors ipsilaterally to treatment area. Changes in crew seating and task allocation to ensure non-traumatic fall over to treatment area. Plan B discussed.
Short monitor cords prevented turning the ECG monitor towards the person assessing the collapsed patient.	Relocation of the ECG monitor, AED, and staff emergency posts within treatment area. Monitor cording improved. Plan B discussed.
Delay in finding the telephone for an emergency call.	Telephone kept in standard position. A staff member designated in charge of the telephone. Relevant telephone numbers mounted in strategic places around the lab, ensuring they are visible from treatment area also.
Uncertainty of the exact address of the lab facility during the emergency call. Describing the different access points required some concentration.	Printouts with institution name, coordinates, street address, room, and location map created. Printouts mounted in strategic places around the lab, ensuring they are visible from treatment area also.
The presumed EMS pathway from outdoors to the lab has a section that is too narrow for gurdeys.	Appropriate pathway established. Alternative pathway established. Crew trained.
Doors of the alternative EMS pathway are locked. Difficulty in identifying the appropriate keys. Attending to the problem removes 1 staff member from the BLS team. Risk of getting locked out, which would relocate another staff member.	Appropriate pathway established. Alternative pathway established. Crew trained. Relevant keys kept in standard position. A crewmember designated in charge of EMS call, door opening task, and guiding EMS through the building.
AED patches are the type that require removing ECG leads. In a deteriorating patient-scenario, this means a trade-off between being able to monitor development of the cardiologic status and being prepared for immediate defibrillation.	Different type of AED patches obtained. Backup patches obtained.
Patient profusely sweaty from ergometer strain. May compromise optimal performance of AED.	Towels (and razors) at a hands reach in treatment area.
Time delay between collapse and defibrillation.	AED placement, patch attachment, placement of accessories (towels, razors) within the treatment area checked every day. AED cover detached (adhering to operating manual).
No closed-loop communication.	Non-technical skills discussed.
Overlapping actions.	Specific tasks during an emergency allocated for each staff member. These are revised and orally confirmed daily prior to initiating each experiment. BLS algorithm visible from all angles of treatment area.

Abbreviations: *AED* = automated external defibrillator, *ECG* = electrocardiogram, *EMS* = emergency medical services

Prior to assessing physical capacity, one of the participants indicated having symptoms of angina pectoris during the preceding two weeks and was instructed to consult a MD, after which he received clearance to participate. During the actual tests, one assessment was terminated before and three tests at maximal exhaustion because of ST-depression exceeding 2 mm. For the reminder of the participants, no exceptional symptoms or findings were detected during the exercise tests or health interview.

### Emerging health issues during the controlled experiments and follow-up

Prior to the experiment one participant experienced a sudden drop in blood pressure while preparing for the measurements and was instructed to lie down. After a while, his status was good and he successfully completed the experiment. Furthermore, the heart rate of one participant dropped to 33 bpm prior to the controlled experiment. He was encouraged to seek immediate care and consult a medical doctor. He successfully participated in the experimental measurements without complications at a later stage.

In the course of the experiments a few situations occurred where either the objective measurements, or the subjective symptoms of the participant guided the researchers to implement measures to eliminate possible adverse health effects (Table 3). Only one participant was guided to consult a MD and all other situations could be dealt through consulting the MD on call. Three of the patients were instructed to seek medical care because of BP levels exceeding the recommended levels for awake time ambulatory measurements (BP 135/85 mmHg). For one of the patients the dose of antihypertensive medication was lowered later because of low BP during the measurements.

**Table 3:** Objective and subjective findings of the participants and responses during the experiments involving submaximal exercise in the cold

Objective finding	Symptom of participant	Safety measures
Ventricular irregular HR of one participant for a few minutes while walking in a warm environment. The measured BP during this period was 76/46 mmHg and when repeated 111/53 mmHg. The RR interval, however, did not deviate from the values measured during the reminder of the experiment.	No symptoms	The participant was instructed to consult a MD because of low BP during baseline rest. A low BP needs to be considered because of post-exercise hypotension.
One measurement conducted while exercising in the cold was terminated because of ST-depression exceeding 2 mm and detecting adjacent ventricular ectopic beats.	No symptoms	The ECG returned to normal after cessation of exercise.
Four participants experienced post-exercise hypotension. Three of these occurred after walking in a warm environment (BP 60/35 mmHg, 68/35 mmHg and 87/50 mmHg) and one after exercise in the cold (BP 87/59 mmHg)	Nausea, dizziness	Participants were instructed to lie down until BP recovered to normal. This occurred briefly, after ca. 5 min.
A total of six participants experienced milder post-exercise hypotension (systolic BP < 100 mmHg). In four cases these occurred after exercise in warm and for two participants in a cold environment.	No symptoms	Constant monitoring the responses and inquiries of subjective symptoms. No additional measures taken.

## Discussion

The current study describes the implementation of safety practices in a novel controlled study design involving cardiovascular strain among a susceptible population of cardiac patients. It is well known that both cold exposure and exercise separately stimulate cardiac function, but their combined effects are not well known. Persons with CAD may be at higher risk while exercising in a cold environment due to the higher cardiac strain and simultaneously reduced myocardial blood flow. Especially studies employing sustained submaximal exercise recommended for secondary prevention in CAD [11] in a cold environment have been lacking.

We followed the ethical guidelines for medical research involving human subjects [1] and paid additional attention for anticipating and reducing any health risks of the participants. For this purpose, the entire research team carefully determined the inclusion criteria for participation. We decided to select stable, relatively healthy and asymptomatic CAD patients for the study. Following this, eligibility to participate was confirmed through different phases, first by the cardiologist, and later by the project personnel (researchers, exercise physiologist, MD, emergency nurse) through further screening of health and assessment of physical capacity. Safety measures for conducting the physical capacity test included using a risk assessment tool [15], and based on a simulation, planning the required response. The controlled experiments themselves included planning and implementing agreed termination criteria, as well as constant monitoring conducted by the researchers, emergency nurse and MD (on call). Finally, the research team ensured recovery of cardiovascular responses immediately after and through 24-h monitoring of BP.

The beforementioned procedures enabled us to successfully carry out the comprehensive controlled study and without any major adverse health events. The simulation with risk assessment in association to the physical capacity tests revealed risks to coherent actions that could have been left unnoticed otherwise. This also underpins the need for emergency response training of any project personnel [14] that are associated with controlled research involving human subjects. The experienced drop in HR or low BP before the controlled experiments required attention by a MD, but enabled successful participation at a later stage. During the experiments exercise in the cold resulted in myocardial ischemia (ST-depression exceeding 2 mm) and termination of the exercise in one patient. This response was expected due to the higher myocardial oxygen demand of cold and exercise, and lower supply of patients with CAD [7, 8]. In addition, post-exercise hypotension and related symptoms were observed both after exercise in a cold and neutral environment in a few patients. Also these responses were expected and allowed planning of the required actions. Hence, these could be resolved on site and enabled continuation of the experiment. To our knowledge, this is also the first study involving cold exposure and exercise with a lengthy follow-up. We detected no abnormal signs in BP or ECG either immediately or 24 h after the experiments.

### Strengths and limitations

The strengths of this study include a comprehensive description of the various phases of planning and implementation of a controlled study involving cardiac stimulation of CAD patients. The controlled study itself involved strict control of both the level of thermal exposure and exercise. Furthermore, each subject served as his own control, through participating in all of the four different experimental conditions eliminating confounding due to intraindividual variation. Strengths also included special consideration of the safety of the patients, with emphasis on pre-screening potential risks, executing emergency simulation for the staff, emergency task allocation, devising a protocol for internal and external communications during an emergency, involvement of the local EMS before the study, and securing unrestricted access point to EMS during an emergency. A limitation of the study is that it is restricted to presenting the implementation of patient safety in a descriptive manner.

## Conclusions

Appropriate beforehand design is a key to implementation of controlled studies involving cardiac patients and augmented cardiovascular strain. Specific factors contributing to patient safety includes careful selection of participants, having sufficient and knowledgeable staff, as well as identifying possible emergencies and designing the required responses. Sharing information of patient safety promotes discussion on the need for more structured safety measures in experimental studies and clinical trials that include potentially hazardous interventions or exposures. Future research needs include systematic analyses of detected health outcomes, usability of current risk assessment methods in controlled studies, as well as effectiveness of implemented safety measures. This will increase scientists’ awareness of required responses and aid developing more systematic safety approaches.

## Abbreviations

AED: Automated external defibrillator
ALS: Advance life support
BLS: Basic life support
BP: Blood pressure
CAD: Coronary artery disease
CRM: Crisis resource management
ECG: Electrocardiogram
EMS: Emergency medical services
HR: Heart rate
MET: Medical emergency team
